# Beyond the digital divide: multi-group SEM examination of socioeconomic status, mHealth utilization, and urban-rural physical activity disparities in Indonesia

**DOI:** 10.3389/fdgth.2026.1831481

**Published:** 2026-05-29

**Authors:** Eli Sumarliah, Kawther Mousa, Yogi Iskandar

**Affiliations:** 1 Informatics Department, Al-Ihya Islamic University of Kuningan, Kuningan, West Java, Indonesia; 2 Public Health Department, Al-Ihya Islamic University of Kuningan, Kuningan, West Java, Indonesia; 3 Institute for Research and Community Service-LPPM, Al-Ihya Islamic University of Kuningan, Kuningan, West Java, Indonesia

**Keywords:** digital divide, Indonesia, mHealth utilization, multi-group SEM, obesity, socioeconomic status, urban–rural disparities

## Abstract

**Background:**

Mobile health (mHealth) technological innovations are now widely being promoted as a scalable solution to the rising problem of obesity. Unfortunately, there is a dearth of empirical research on the extent to which mHealth utilization is associated with acceptance disparities in emerging economies.

**Objective:**

This work explores the associations among socioeconomic status (SES), urban-rural differences, mHealth utilization, and sustained physical activity in obesity management in Indonesia. The paper also assesses urban–rural variation in the associations among SES, mHealth utilization, and sustained physical activity.

**Methods:**

Quantitative assessment was used to examine the direct and indirect associations between SES, mHealth and sustained physical activity among 1,204 overweight and obese respondents in Indonesia. Multi-group SEM (structural equation modelling) was performed to assess differences between urban and rural cohorts and to examine group differences between the two samples.

**Results:**

The empirical outcomes indicate that (1) SES is significantly associated with mHealth utilization and sustained physical activity, (2) mHealth utilization is significantly related to sustained physical activity for obesity control, and (3) Structural differences exist between urban and rural groups in the strength and significance of the associations among SES, mHealth utilization, and sustained physical activity among overweight and obese adults.

**Discussions:**

The empirical outcomes align with earlier publications that discovered a substantial linkage between SES and mHealth utilization. The outcomes showed a positive correlation between SES and sustained physical activity, differing from an earlier work. This may be attributed to contextual differences: prior cohorts with higher wealth engaged in sedentary lifestyles, while here the link of SES-exercise was positive due to greater access to resources. Additionally, while earlier research has suggested mHealth utilization h as a psychosocial mediator, our outcomes indicate geographic disparity, where the benefits of technology are linked to urban infrastructure. It implies that mHealth utilization may be more prevalent in high-SES Indonesian cities, potentially widening health disparity gap.

## 1 Introduction

One of public health concerns that has been progressively identified as significant in emerging economies is obesity (1–4). Elements that have led to a buildup in the prevalence of obese and overweight individuals are rapid urbanization, changes in dietetic practises, and inactive way of life (3–5). However, these issues are not evenly distributed and are influenced by socioeconomic and geographical disparities (3, 6). On a global scale, digital health, particularly mobile health (mHealth), has been promoted as an effective and cost-effective approach in handling protracted ailments (1, 7). In Indonesia, the digitalization initiatives are associated with increased use of telemedicine, fitness trackers, and health monitoring apps (1, 8) but digital access’ availability is not equal (9, 10). Internet penetration, smartphone use, and internet speed vary considerably between urban areas like Jakarta and rural provinces in eastern Indonesia (8, 11). This has sparked apprehensions regarding the “digital divide” and its association with health equity (7, 12).

However, some research gaps remain exist. Although a great deal of literature has explored the social determinants of obesity (1, 13, 14), relatively less literature has explored the digital infrastructure aspect of structural inequality (12). The existing publications on mHealth in emerging economies has largely been concentrated on (a) the effectiveness of particular behavioral interventions, particularly in terms of short-term weight loss, such as (1, 15), (b) technological feasibility, e.g. (7, 16),, and (c) user enjoyment outcomes (17–19). There is little investigation into the potential for digital health interventions to systematically favor individuals of higher socioeconomic status (SES) and urban dwellers over rural and lower-income communities (20). There is evidence that digital healthiness interferences have a tendency to bolster persons of upper SES and urban dwellers, a trend that has been termed the “third digital health divide” (7, 12). Although digital health can increase access, it may also be linked to widening existing health disparities because individuals of higher SES tend to have the necessary devices, internet access, and eHealth literacy to effectively use these technologies (7, 20).

To tackle these gaps, this paper evaluates digital health in relation to obesity, conceptualizing it as more than a neutral behavioural technology. It builds and validates a multi-level socio-technical framework to probe the function of socioeconomic aspects, urban-rural infrastructure, and global virtual governance in influencing mHealth utilization, physical activity and obesity in Indonesia. Furthermore, digital health interventions are marketed as cost-saving approaches to obesity, presented as neutral technologies for individual behavior change (1, 5). This work challenges this premise and suggests that the healthcare technology industry may be related to sustained physical activities for obesity management and broader health outcomes, with potential implications for health inequalities. This study challenges health inequalities in the digital age by looking beyond income, education, and governance to digital infrastructure, internet access, and technological literacy. This work represents one of the initial attempts to conduct a multi-group examination of the association between mHealth utilization and health inequalities in relation to sustained physical activity for obesity-related outcomes in Indonesia. The query to be resolved as the final aim of this research is whether the digitalization of healthcare is a contributing factor to the reduction of health inequalities or the widening of structural divides in emerging economies.

Below are three research queries (RQs) to be addressed:

RQ1: How are SES, mHealth utilization, and sustained physical activity related among obese and overweight individuals?
RQ2: Does mHealth utilization mediate the association between SES and sustained physical activity?
RQ3: Do the indirect associations among SES, mHealth utilization, and sustained physical activity differ between urban and rural cohorts?

To answer those RQs, this work employs multi-group SEM for hypotheses assessment. The empirical outcomes reinforce the progression of high-grade publications and reveal whether digitalisation alleviates or perpetuates structural health inequities in the emerging nations. For this research, mHealth utilization (MHU) can be described as how often a person uses mobile apps for their healthcare and SMS-based health interventions to keep track of their physical activities and encourage them to achieve health-oriented objectives. Sustained physical activity (SPA) is a term that describes the constant efforts of maintaining fitness through exercising, and measured by self-perceived habit strength and reported activity levels.

## 2 Review of literature and hypotheses

### 2.1 Groundwork theories

Our paper employed the insights of the Digital Divide Theory, Social Determinants of Health (SDH) approach, and behavioral theories of technology-facilitated health behavior change to address the issue of disparities in mHealth-supported physical activity. The SDH approach has emphasized the influence of numerous structural elements on health behavior and health outcomes (13, 21, 22). Structural factors, consistent with SDH approach, affect the accessibility of healthiness resources and the capacity to perform preventive health behaviour (13, 22). Though digital health solutions can be regarded as an intervention strategy at the personal level (1, 17–19), this work attempts to investigate how social determinants such as socio-economic condition and geographical location may relate to the effectiveness of mHealth utilization in Indonesia through the lens of SDH.

In terms of digital health, disparities in socioeconomic status are reflected as disparities in the accessibility of technology and health information (7, 12). The Digital Divide theory emphasized the contribution of technology to the continuation of existing disparities between societal groups, which are reflected as disparities in access to technology and health information (7, 23). The mHealth utilization was found to be dependent on various socioeconomic and geographical conditions (6, 19, 20). This approach, however, also points to the need to explore the possibility that health inequalities may be addressed via digital wellbeing interventions (12).

At the behavioral stage, the Social Cognitive Theory offers alternative theories to explain the continuing physical outcomes of mHealth utilization (5, 24). Self-monitoring, feedback, and goal-setting capabilities enabled by mHealth utilization may promote self-efficacy and intrinsic motivation to engage in physical activity, which may result in long-term behavioral outcomes (5, 24). Through amalgamating the structural and behavioral approaches, the paper developed a theoretical framework in which mHealth utilization may mediate the relation between socioeconomic resources and long-term physical outcomes as a socio-technical mediator, as proposed in the arguments presented in earlier works (19, 20). The proposed theoretical framework of the paper also suggests the anticipated associations of socioeconomic and spatial inequalities with sustained physical activity for obesity management through interventions reinforced by mHealth utilization, following an earlier publication (6).

### 2.2 Hypotheses formulation

#### 2.2.1 SES and mHealth utilization

SES (socioeconomic status) is among the most reliable forecasters of health-linked activities and care services (19, 20). The Digital Divide Theory solicits “discrepancies in accessibility and skills with technology mirror existing social inequalities” (12, 23). Hence, mHealth utilization will not be socially neutral but may show a relation with differences in resources and capabilities due to SES, as reported in existing publications (12, 20, 23).

It has been recognized by academicians that people who belong to a high SES have more opportunities for accessing mHealth due to their economic strength, digital literacy, and connectivity. For instance, as per the study by Price-Haywood et al. (7), although smartphones have become prevalent with everybody, economically disadvantaged classes suffer from inadequate broadband connectivity, lack of service availability, and no insurance cover, which inhibits them from making effective use of their smartphones for mHealth utilization. In addition, there is also an evident related to the digital divide across rural–urban environments, gender, and health conditions, which highlights continuing gaps in accessibility, usage, and digital knowledge., as observed by Zhang et al. (12).

In addition, education boosts mHealth utilization by improving health literacy and competence in applying digital health information. The lower socio-economic status groups generally face double constraints in terms of these competencies and access to information, as Brennan et al. (20) advised. Additionally, the findings of Azam et al. (14) suggested that mHealth utilization is influenced by intentions to act, self-efficacy, and usefulness, which are linked to SES. Despite providing enhanced health management and quality of life benefits, mHealth utilization is not equally effective when these disparities are left unaddressed, as highlighted in earlier works (19, 20).

The link between SES and mHealth utilization can be seen through access, digital skills, and health literacy; consequently, people with high SES may use mHealth for maintaining physical activities for combating obesity. Therefore:

**H1:** Socioeconomic status is positively associated with mHealth utilization for sustained physical activity.

#### 2.2.2 Mhealth utilization and sustained physical activity

The linkage between mHealth utilization (MHU) and sustained physical activity (SPA) is regarded as being less direct and intricate than before. This is because MHU is not acting as an independent motivational tool but rather as an instrument which leverages behavioral and cognitive processes so that people can perform SPA. Earlier publications have advised that mHealth apps can enable personal monitoring and awareness of behaviors in encouraging physical activities. For instance, academicians claimed that digital tracking of physical activities and its associated behaviors have disclosed great compliance; however, without more encouragement, this compliance may be reduced (15, 25). Another group of researchers (25) argued that one's continual engagement is essential for the SPA, among other things such as feedback and intervention that reinforce goal pursuit and self-regulation.

Interventions based on theoretical approaches reveal the significance of psychological constructs. For example, an earlier publication (5) discovered that the diversity of MHU such as the use of applications for fitness together with social networking sites has been successful in improving SPA behavior mostly due to increased self-efficacy and social support. In addition, due to health services associated with telehealth counseling, MHU can be strengthened and subsequently SPA can be more easily adhered to (1).

Furthermore, mobile and wearable tools can substantially relate to SPA because of their capacity to provide instant feedback and accessibility improvements to enhance adherence, as argued by researchers (24, 26). However, another group of scientists (27) observed that this kind of correlation is not always reliable because when user engagement weakens, some interventions have limited long-term influences. Furthermore, other factors like social and environmental influences can play a role in the use of mHealth and physical activities, as exposed by other experts (13, 28).

In the end, user satisfaction in MHU become a critical factor in ensuring its meaningful relation with SPA since high quality systems and interaction guarantee that the mHealth apps remains useful to individuals, as demonstrated by scholars (17). Generally, MHU becomes meaningfully related to SPA behaviors; yet, various factors contribute to its intensity. Therefore:

**H2:** The mHealth utilization is substantially linked to sustained physical activity.

#### 2.2.3 SES and sustained physical activity

The link between SES and SPA is rather complicated. This association may manifest in a direct and indirect way in accordance with certain mechanisms like via accessibility to resources, societal conditions, and structural constraints that may form continuing engagement. A number of sources (29–31) ascertain that people from low SES tend to involve themselves in inadequate levels of physical activities due to various financial, environmental, and access-related aspects. Nevertheless, the correlation between SES and SPA cannot be regarded as uni-directional. On the contrary, high SES and affluence might negatively relate to SPA in specific situations, as a report (32) claimed.

An earlier work (6) disclosed that the SES-SPA link is done through social support and lifestyle mediation, which could influence behaviors and health outcomes. Meanwhile, another work (21) reported that other aspects of social determinants such as education and earnings may help boost the chances for people to perform SPA (21). Other facilitating aspects, including those related to contextual attributes such as gender, age, and culture, add to the physical activity inequalities gap, as a published paper (31) unveiled. Generally, SES is a variable that plays a vital but indirect role in physical activity involvement, which is shaped by many other aspects, as advised by academics (29).

As it can be noted from the review of literature, there is evident interconnection between SPA and SES. The reason for such interconnection is that owing to certain resources, social conditions, and privileges provided by SES, people have an opportunity to be involved in SPA, i.e., maintain regular exercise over time (29–32). Therefore:

**H3:** Socioeconomic status is substantially linked to sustained physical activity.

#### 2.2.4 Mediation of mHealth utilization

An earlier empirical work verified that mHealth utilization mediates the psychosocial features and health outcomes (33). Considering the fact that SES affects digital engagement and exercise behavior (29, 30, 34), mHealth utilization could be an intermediary between SES and exercise behavior (20, 35). The Digital Divide model states that persons with higher SES have more chances of using digital tools to manage their health (12, 23). These factors can potentially enable individuals to use their SES resources to improve their health behaviors through digital engagement. Meanwhile, the SDH theory indicates that healthiness conducts could be influenced by structural factors through numerous pathways (22). Considering the fact that SES also influences the physical environment for exercise behavior, as suggested by empirical reports (20, 31), fractional mediation is expected. Hence, mHealth utilization can act as a vital behavioral mediator through which the obesity-related outcomes are influenced by SES inequality.

**H4:** mHealth utilization mediates the relation between socioeconomic status and sustained physical activity.

#### 2.2.5 Urban–rural differences

Spatial inequalities have an important position in physical activity and mHealth utilization (12, 21, 36). The Socioecological theory highlights the environmental component in the adoption of health behavior through the availability of infrastructure and resource apportionment (37, 38). Urban areas usually have better telecommunications infrastructure, fitness centers, and exposure to digital healthiness promotion campaigns (37). Those factors may affect mHealth utilization and physical activity engagement (8, 12, 27).

On the contrary, there might be limitations in connectivity, recreational facilities, and health promotion programs in the rural areas (12, 37). Based upon the Digital Divide Theory, the spatial inequities could cause the development of differences in the efficiency of technological access and mHealth utilization, which has been documented in earlier works, e.g. (7, 12, 23). The spatial differences in the aforementioned contexts indicate the potential variability in the structural relations amongst SES, mHealth utilization, and sustained physical activity. Hence:

**H5:** Structural relations amongst SES, mHealth utilization, and sustained physical activity differ between urban and rural populations.

### 2.3 Conceptual framework

**Figure 1:**
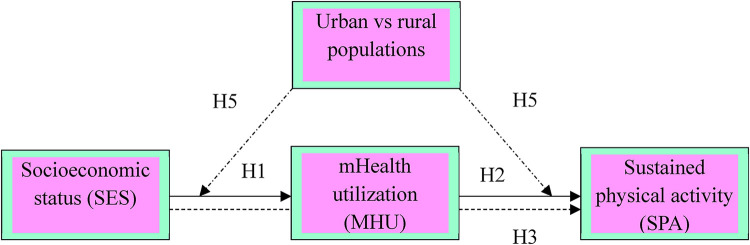
Conceptual framework.

The hypothesis creation brings about conceptual framework outlined in Figure 1.

## 3 Methods

### 3.1 Data gathering and respondents

The work was carried out following cross-sectional scheme advised by Oetoro et al. (1) to scrutinize socioeconomic and spatial variations in mHealth utilization for nutritional obesity management in Indonesia. Data collection for the research was carried out from 7 to 27 February 2026, wherein a set of questions was administered to adults classified as overweight or obese according to their personal reports concerning weight and height measures. BMI (body mass index) was computed according to standard criteria, wherein a BMI of 25 or higher was considered for inclusion in the study (1). Responders must be at least 18 years of age and must be capable of independently completing the set of questions, as those who are not capable of providing reliable self-reported data were excluded from the study. The questionnaire was based on the socioeconomic factors, continual physical activities, and mHealth utilization. It utilized validated measures, which were pilot-tested on 40 samples prior to the full-scale research. The questionnaire used Indonesian language.

The proportionate stratified sampling design was applied in this research as suggested by earlier works (37, 39), taking into account the urban-rural proportion of the population in Indonesia, encompassing multiple provinces and various digital infrastructure and socioeconomic progression. The strata considered in this work are as follows: (a) The urban inhabitants, which designate individuals dwelling in the *kota* (municipalities), and (b) The rural inhabitants, which specify people residing in the *desa* (villages). The concept of urban and rural is based on the classification made by *Badan Pusat Statistik,* a.k.a. Indonesia's Central Bureau of Statistics (9). A ‘rural’ area (*desa)* is an area where more than 50 percent of its residents are occupied in agriculture and other informal sectors, and has low density and lack of facilities. An ‘urban’ area (*kota*) is a regency/city where more than 50 percent of its residents are engaged in non-agriculture occupation, and has high density, greater infrastructure and facilities. Indonesia consists of different peri-urban and small town areas; nonetheless, in this paper, special consideration was paid to selecting respondents from only rural and urban zones. The motive is to make the study more internally valid and distinctive regarding infrastructure, digital access, and lifestyle. Besides, the binary categorization delivers the biggest possible differentiation in structures and socio-environment applicable to mHealth utilization and sustained physical activity among the two distinct cohorts**.** For multi-group SEM purposes, it is imperative to classify the area into two categories to ensure an adequate number of respondents in each group. Based on data from 2025, 40.5 percent of the Indonesian population reside in rural areas while 59.5 percent of them reside in urban areas (11). The allocation of sample in this research is geared toward ensuring that there is proportionate representation of sample units in accordance with the abovementioned ratio of the urban to rural areas among the entire population of the country. This is to make sure the adequate power for multi-group SEM comparisons.

In each stratum, respondents were chosen through a combination of approaches that are geared towards ensuring robustness, validity, representativeness in research findings (40): (a) Offline/direct recruitment from *Puskesmas* (community health centers) and local organizations, and (b) Online recruitment via social media, for example, WhatsApp, the most popular messaging app in Indonesia (41), which was targeted to community groups. Respondents were selected through a quasi-probabilistic approach within stratum because participation depended on individuals’ voluntary willingness and access to specific community groups (42). The research was carried out voluntarily, and the responders were asked for their consent prior to collecting information. No personal identifiable data was included in the dataset to safeguard confidentiality. The work obtained authorisation from Research Ethics Committee within Al-Ihya Islamic University of Kuningan (Reference number: AIU-REC/2026/II/024). It means that the investigation was carried out based on the ethics guidelines.

Methods of duplication prevention are as follows. For offline/direct recruiting, enumerators were specially trained to check if respondents met all the recruitment criteria and to track their survey completion. For online recruiting through WhatsApp, (a) unique identifiers was given to every participant; either system-generated or enumerator-generated (respondent code) to avoid duplicate entries, and (b) screening questions (e.g., prior participation confirmation items) were asked to detect and flag potential repeat submissions. In total, there were 1,356 filled out questionnaires, but only 1,204 could be analyzed after data cleaning, which means the usable response rate was around 88.8% with a greater proportion of respondents from urban locations because of their high accessibility. It is unreasonable to give a precise rate of responses owing to the use of quasi-probability sampling method during the research process.

Furthermore, the problem of missing data was addressed through the method of pairwise deletion in correlation analyses and FIML (full-information maximum likelihood) in SEM (43). Excluded respondents were those with: (a) duplicate submissions (*N* = 12); and (b) incomplete or invalid responses, including those with more than 50% missing data (*n* = 140). The final analytical sample was composed of 1,204 respondents: 706 (58.6%) urban populations and 498 (41.4%) rural populations, which is close to the national demographics of Indonesia i.e., 59.5% urban and 40.5% rural (11). This sample was satisfactory for multi-group SEM examination (39, 44).

### 3.2 Measures

#### 3.2.1 Mhealth utilization

To measure mHealth utilization, we used six items (MHU1–MHU6), where responders signposted their agreement using five-point Likert-type response format: 1 = fully disapprove, 2 = disapprove, 3 = neutral, 4 = approve, and 5 = fully approve. Responses were averaged across items to compute an overall mHealth utilization score for every responder, such that bigger values denoting greater mHealth utilization for sustained physical activity and obesity management**.** The five items (MHU2–MHU6) were validated items adapted from earlier works (1, 14). Meanwhile, the first item (MHU1) stating that “Using mHealth applications motivates me to maintain regular physical activity” was newly created by the authors and later validated through assessment from 10 experts before gathering data (see Supplementary Appendix Table A6 column 2 & Questionnaire Part 1 for the complete measurement items). The Cronbach's alpha (*α*) estimates were .85 for the rural subsample and .88 for the urban subsample, signifying tolerable internal consistency for the two cohorts (Table 4). For MHU construct, it employs a reflective method for the measurement of usage, usefulness, and motivation, which gauges the person's tendency towards using the mobile application for mHealth purposes. Even though there have been some behavioral and attitude indicators employed within the instrument, technology adoption and mHealth utilization have been closely related to both behavioral and perceptual measures (45). For all six MHU measures, loadings were very high in both urban (*λ* ≥ .77) and rural (*λ* ≥ .74) areas, which reinforces the latent construct's unidimensionality. The mHealth utilization scale demonstrated acceptable psychometric properties in both samples. This is evidenced by the fact that Cronbach's alpha and composite reliability scores exceeded the necessary cutoff level, which is enough to certify instrument's validity and reliability.

The survey focused on the universal mHealth applications that are used to monitor the health of individuals and their physical activities. Some of the popular health services applications widely used by Indonesian responders include Halodoc, Alodokter, Mobile JKN, and SATUSEHAT Mobile, and they help people manage their health via consultation services, reminders, and basic monitoring functions like goal setting and habit tracking. The examples of other fitness tracking applications include Strava, Mi fitness, and Huawei Health, which act as activity trackers, step counters as well as counter of energy expenditure by the users. The survey responders also utilized applications for healthy living management and exercise such as FITA and Yes.Fit, which also help one monitor the food intake as well as body weight goals. The survey did not restrict responders to using any particular brands or apps; thus, their reactions reflected general engagement with the mHealth technologies instead of their personal experience with a particular brand.

The reason why the functionality aspect of the mHealth application was highlighted rather than its category is that it enhances the generalizability of the findings and becomes easier to implement the results in various cities and villages in Indonesia. Moreover, because the research method considers the frequency of usage of the mHealth apps and the perceived function of mHealth tools on health behavior, the measurement is considered reliable, comprehensive, and valid in representing the latent construct of mHealth utilization within the SEM framework.

#### 3.2.2 Sustained physical activity

Sustained physical activity (SPA) was computed by a reflective multi-item scale that measured the respondents’ habitual and consistent involvement in physical activity behaviors that are pertinent for obesity control. Five items (SPA1, SPA2, SPA3, SPA4, and SPA5) were constructed by the researcher based on pilot interview findings with experts in public health and physical activities and a bibliometric examination of physical activity publications for content validity and context-specificity for obesity control. An example of a scale item is: “I engage in physical activity regularly every week,” (see Supplementary Appendix Table A6 column 2 & Questionnaire Part 2 for the complete measurement items). The responders’ agreement with the scale items was gauged on five-category Likert measure, from 5 (fully approve) to 1 (fully disapprove), with the higher score designating more consistent physical activity. Physical activity levels were quantified by the adjusted Saltin-Grimby scales [Gottfridsson et al. (46); Lennartsson et al. (47)], which calculated the responders’ physical activity levels for the past year in their leisure time and other activities (including transportation-linked activities). The item (SPA6) was appraised on a scale from 1 (almost inactive) to 4 (highly active).

The measurement of SPA has five items based on Likert scale and one item based on the four-category Saltin-Grimby scale. Explanation as to why this measurement is used is as follows: (a) Each group uses the same sets of items in measuring the same tendency to engage oneself in physical exercises which consist of frequency, consistency, and intensity; (b) The factor loadings of .75 ≤ *λ* for all the items used in SPA have been found to be good in the CFA, showing that the question taken from Saltin-Grimby objective was congruent with self-reported daily activities; and (c) The AVE and CR of both rural and urban cohorts were good and thus reinforce construct validity.

Further experiments were executed to make sure the measurement framework's robustness. First, convergent validity was verified in the model since all the constructs had average variance extracted (AVE) above .60, which surpassed all the threshold values. Second, internal consistency was ascertained since the composite reliability value was between .83 and .90, which implies strong reliability across cohorts. Lastly, discriminant validity among constructs was supported by conducting the Fornell-Larcker Criterion test, which exposed that constructs had empirical distinction.

#### 3.2.3 SES

SES (socioeconomic status) has been reflected as an underlying construct based on three indicators: educational level (SES1), household income (SES2), and job stability (SES3) of the responders (see Supplementary Appendix Table A6 column 2 & Questionnaire Part 3 for the complete measurement items). These indicators were selected based on the validated measures from an earlier (6) except for social class identity which, in this paper, is replaced by job stability. Such substitution is justified as job stability delivers a more objective, structurally relevant, and context-sensitive indicator of SES, whereas social class identity is subjective and potentially less reliable in measuring actual socioeconomic conditions.

The level of education was computed with four categories based on national classification by the Central Bureau of Statistics, Indonesia (9). The categories included higher education, upper secondary schooling, lower secondary, primary education and below. Additionally, individual households’ earnings were determined by the sorted income ranges based on the national poverty statistics by *Badan Pusat Statistik* (the Central Bureau of Statistics), Indonesia (48). As per March 2024, the line of poverty for each household was IDR 2,786,415 monthly. Hence, the responders were classified into five groups of the monthly income: less than IDR 2.8 million (poor), IDR 2.8–5 million (lower income), IDR 5 million to 8 million (lower-middle), IDR 8 million to 12 million (middle), and more than IDR 12 million (upper).

The measurement of employment stability is based on the main employment status and income regularity of the responders and is divided into four types: (a) being unemployed or having an irregular work pattern, (b) having an informal job with an unstable income, (c) being self-employed or having small business with moderately stable income, and (d) having formal employment with regular income. The higher the SES scores, the greater socioeconomic advantage. The paper did not employ Fan et al. (49)'s scale as it is based on the perception of employment condition, while this work aims to measure it as one of the objective aspects of SES so that urban-rural comparability included in the SEM assessment can be ensured.

The SES is computed from the following three measures: education (SES1), household income (SES2), and occupational stability (SES3) using standard multivariable indicators of SES in connection with public health and social epidemiology (50, 51). Employment stability can have different operation in urban and rural areas. To solve this problem, (a) Multigroup SEM is used to compute the factor loadings for both cohorts while ensuring that the latent variable SES incorporates common variability of the items while at the same time allowing for context-specific variability, and (b) The factor loadings of employment stability were revealed to be significant for both cohorts (*λ* = .71 rural, *λ* = .74 urban), which suggests its meaningful contribution to the total SES construct regardless of market dissimilarities.

In the SEM approach, if the studied variable is a latent variable on account of its method of construction, then in the construction of this variable, all the three variables contribute equally according to their factor loading, whereas in any other framework where there is an assumption of equal weighting, all the three contribute equally in construction of this variable. In this research sample involving urban residents, factor loadings of variables such as education, income, and occupation stability were .78, .82, and .74, respectively. It means that education was highly significant, although not dominating the construction of the variable. This was proven through sensitivity examination with a new framework wherein education is a sole indicator. It can be noted that both SEM paths related mHealth utilization and sustained physical activities had statistical significance; thus, it is clear that the detected inter-variable links was not merely triggered by education but by the amalgamation of several SES elements.

All indicators used in this paper were thoroughly assessed using the criteria of their item loadings, cross-loadings, average variance extracted (AVE), and composite reliability (CR). To ensure that an indicator is not discarded during further analysis, the following conditions must be met: (a) factor loadings above .70; (b) CR and AVE values must be greater than .80; and (3) theoretical significance to the construct.

#### 3.2.4 Control variables

Some demographic aspects were added as control variables, which were able to control the impact of possible confounding outcomes. They were age, gender, and residential area. Gender was inscribed as twofold component (1 = female, 0 = male), and the residential area was also enciphered as dualistic (0 = rural, 1 = urban). These controllers were added because some earlier publications exhibited that they might affect digital health adoption as well as physical activity (1, 24).

### 3.3 Bias and data quality considerations

#### 3.3.1 CMB

The check for common method bias (CMB) was performed through the common latent factor (CLF) technique within SEM framework. Here, all indicators were assigned to a latent construct that accounted for the variance across different methods. Supplementary Appendix Table A4 comparing loadings with and without a common latent factor (CLF) to show that common method bias is minimal. The difference between standardized coefficients of indicators before and after applying CLF was only .02 or less (*Δ* ≤ .02); therefore, CMB should not be regarded as a source of bias in this research.

Some procedural precautions were adopted to ensure that CMB did not occur. First, promises on anonymity and confidentiality were made to the respondents to ensure that the issue of social desirability and dishonesty were addressed. Second, questionnaire development involved a systematic arrangement wherein items for different constructs (SES, MHU, and SPA) were put in different sections to ensure that respondents would not give similar answers. Finally, changes were introduced to the format of the questions, where most of them contained a five-point Likert scale, while some of them contained categorical questions (e.g., SPA6, SES). This variation is to alleviate the risk of method-induced correlations.

To examine the possibility of CMB, some statistical approaches were adopted. The initial step is executing the Harman's one-factor assessment. As shown from the results of the Harman's test, it can be noted that when adopting the investigative factor examination technique towards the entire variables, the initial factor did not account for most of the variances since it only 38 percent of the variances. It means that CMB does not appear to be an issue here. Second, the SEM analysis included the examination of a latent common variable for the purpose of evaluating the influence of the overall method factor on the correlations established. Based on the data obtained, the method factor had minimal effect on the variance (below 5%).

#### 3.3.2 Self-report bias and measurement limitations

The researchers realize the risk of causing the responders to either underreport or overreport their mHealth utilization behaviors. Particularly, the subjective self-reported data that was obtained from the participants about their heights and weights could have been subjected to issues related to recall and social desirability bias. The potential bias might have influenced inclusion criteria and dependent variable, as well as resulted in measurement errors and, consequently, affected the hypothesized correlations. Hence, this problem was mitigated by ensuring anonymity, having clearly described methods of frequency measurement, and measuring method variance using *post hoc* statistical examinations. Moreover, in this paper, structural equation modeling (SEM) with robust assessment approaches was applied to eliminate bias in parameter estimates due to minor measurement error (52). Besides, although self-reported data can carry some biases, there are earlier publications demonstrating that self-reported physical activity is a valid measure for large-scale examination on behavior patterns [e.g., (53)]. Moreover, to ensure that the data was accurate, we instructed the responders on how to report their information and gave them examples. Sustained physical activity (SPA) assessment was conducted with the help of a self-reported questionnaire that may have resulted in the distortion of information about the actual SPA level of respondents. For example, respondents might not perceive occupational or incidental physical activity as exercise. Thus, the survey encompassed questions on all the three domains of physical activities which include exercise for recreation, commuting to work by walking or cycling, and occupational activities at home like simple gardening (see Supplementary Appendix Questionnaire Part 3, SPA6).

### 3.3 Analytical approach

In the current work, the authors develop SEM (structural equation modeling) methodology which inspects the associations among socioeconomic status (SES), mHealth utilization (MHU), and sustained physical activity (SPA) in urban and rural regions, wherein some sociodemographic controls are performed. In doing so, the SEM involves testing for direct and indirect relations to determine the mediation role played by MHU in the link of SES and SPA. Besides, a multigroup SEM will also be operated to identify whether there is any difference in these relations within urban and rural cohorts in Indonesia. The hypothesized links are tested by means of the SEM where structural and measurement frameworks are estimated simultaneously, as well as mediation and group differences are assessed within an amalgamated analytical approach.

Mathematical equations to assess hypotheses are as follows:

For direct associations in hypotheses 1, 2, and 3 (H1–H3), let:

Age,Gender,Residence(Urban=1,Rural=0)becontrolvariables

The first hypothesis (H1: SES → MHU) is tested with Equation 1:

$$\mathrm{MHU}=\beta_{1}SES+\Upsilon_{1}\mathrm{Age}+\Upsilon_{2}\mathrm{Gender}+\;\varsigma_{1}$$ (1)

For the second and third hypotheses (H2 & H3: MHU and SES → SPA), use Equation 2:

$$\mathrm{SPA}=\beta_{2}MHU+\beta_{3}SES+\Upsilon_{3}\mathrm{Age}+\Upsilon_{4}\mathrm{Gender}+\Upsilon_{5}\mathrm{Residence}+\varsigma_{1}$$ (2)

Here, H1, H2 and H3 are supported when $\beta_{1}$, $\beta_{2}$, $\beta_{3}>0$, separately

For mediating function (H4), use Equation 3, and for total effect of SES on SPA, uses Equation 4:

$$\mathrm{Indirect}\;\mathrm{effect}=\beta_{1}\times \beta_{2}$$ (3)

$$\mathrm{Total}\;\mathrm{effect}=\beta_{3}+(\beta_{1}\times \beta_{2})$$ (4)

Mediation is supported if: $\beta_{1}\times \beta_{2}>0$, and significance was tested via bootstrapping. Partial mediation is attained when: $\beta_{3}\neq 0$, and full mediation: $\beta_{3}=0$.

For multi-group SEM for urban–rural differences (H5), estimate structural paths separately for group 1 (urban) using Equations 5, 6 and group 2 (rural) using Equations 7, 8 as below:

$$MHU_{U}=\beta_{1U}SES+\varsigma_{1U}$$ (5)

$$SPA_{U}=\beta_{2U}MHU+\beta_{3U}SES+\varsigma_{2U}$$ (6)

$$MHU_{R}=\beta_{1R}SES+\varsigma_{1R}$$ (7)

$$SPA_{R}=\beta_{2R}MHU+\beta_{3R}SES+\varsigma_{2R}$$ (8)

H5 is supported if: $\beta_{1U}\neq \beta_{1R}\;\mathrm{or}\;\beta_{2U}\neq \beta_{2R}\;\mathrm{or}\;\beta_{3U}\neq \beta_{3R}$.

Further, test the chi-square difference using Equation 9 where significant $\Delta \chi^{2}$ indicates structural path differences.

$$\Delta \chi^{2}=\chi_{\mathrm{constrained}}^{2}-\chi_{\mathrm{unconstrained}}^{2}$$ (9)

Full structural system is computed with Equation 10:

$$\begin{array}{rl} & MHU=\beta_{1}SES+\Gamma_{1}Controls+\zeta_{1}\\ & SPA=\beta_{2}MHU+\beta_{3}SES+\Gamma_{2}Controls+\zeta_{2} \end{array}$$ (10)

Here, Controls=(Age,gender,residence).

Justification of the multi-group SEM approach is as follows. The issues addressed in this work deals with the associations between SES, mHealth utilization, and sustained physical activity by individuals living in either urban or rural communities, as well as possible differences in such associations across these two cohorts. The application of the multi-group SEM approach will help in comparing the structural paths across cohorts while accounting for measurement errors. In this way, it provides a strong theoretical ground for examining structural models separately in each particular case. Indeed, there might be differences between the studied rural and urban communities with regard to technology accessibility and health-linked behaviors that can shape the role of SES. In other words, the application of multi-group SEM examination has been justified because our study was meant to investigate whether there is any difference in the associations between SES, sustained physical activities, and mHealth utilization between urban and rural locations. Multi-group SEM should be employed when: (i) group-specific differences need to be determined, (ii) spatial moderator roles have to be taken into account, (iii) comparisons of latent structures should be conducted, and (iv) effect sizes must be calculated (44).

The normality test of the univariate distribution was performed through the examination of kurtosis and skewness estimates for the entire constructs. The normality test results showed that all variables had normal distributions due to their skewness and kurtosis estimates being inside the breadth specified (|skewness| < 2 and |kurtosis| < 7) as advised by Kline (54) and Sathyanarayana (55). Mardia's test for multivariate kurtosis was employed to examine whether the data set was normally distributed (55). The outcomes display that multivariate normality was not evident in this case because the issue is quite prevalent in psychological and survey studies (55). In relation to data non-normality, MLR (maximum likelihood with robust standard errors) technique has been used when performing SEM examination (56). Robust MLR technique gives robust standard error as well as robust Chi-Square statistics. This implies that Chi-Square statistics have been adjusted in light of non-normality of data distribution; therefore, obtaining more accurate results of hypothesis testing. Moreover, all bootstrap procedures used to derive confidence intervals for the path coefficients and composite reliability measures have ensured that no potential bias arising from the non-normal distribution of the data set is minimized.

The fit of the framework was estimated via fit statistics of SEM of rural and urban cohorts: CFI > .90, TLI > .90, RMSEA < .08, and SRMR < .08. In addition, indicators’ validity and reliability included in the framework were determined based on the following criteria: Loading of the variables’ items (*λ* > .70), AVE > .50, and composite reliability > .7. Besides, to ascertain the equivalency of the latent variables across the two cohorts, measurement equivalence examinations are conducted. Following Hair et al. (57), three crucial criteria were applied for assessing whether an indicator was adequate. To begin with, an item loading must be greater than .70. Second, it was also necessary that cross-loadings be .20 less than factor loading to establish discriminant validity. Third, all indicators must make positive contributions to construct reliability indices like CR and AVE. This implies that indicators used in this paper are consistent as well as coherent with other constructs, which supports a robust measurement model for the next multi-group SEM assessment.

Measurement invariance must be specified to ensure that digital literacy, mHealth utilization, and sustained physical activity are considered equally across subgroups in both contexts. Multi-group SEM is also beneficial for gauging the comparison of coefficients in paths across contexts, investigating possible moderating roles of geographical location (39). For example, the association between digital access and mHealth utilization may be greater in cities with developed connectivity infrastructure than in rural areas with infrastructural issues. By specifying mHealth as a mediator and moderator of the association between SES and obesity, this study will contribute to understanding whether digital health technologies can mitigate social inequalities or, on the contrary, widen the breach among the poor and the rich. This will ripen evidence-based knowledge about the complex rapports between structural determinants and individual behaviour (21), which offers actionable comprehensions for public health interferences and policy strategy.

## 4 Results

### 4.1 Sample characteristics

Table 1 exhibits the final analytical sample with *N* = 1,204, and confirms a socioeconomic gradient between spatial groups. In addition to education, the other socioeconomic variables used in our study were: (i) Income (SES2) in million IDR, classified as follows: under 2.8, 2.8–5, 5–8, 8–12, and above 12; and (ii) Employment stability (SES3), classified as follows: unemployed/instable job; informal employment/instable income; self-employment/small business/moderate stable income; formal employment/stable income. Table 1 illustrates cross-tabulations between urban and rural population by SES. From our results, it is clear that there is definitely some socioeconomic gradient since urban population consists mainly of individuals having high incomes and formal employment. Hence, the proportion of urban population with low income and informal employment is lower than rural population. High income and formal employment enable people living in cities to buy mobile phones and access internet, as well as find time for exercising. On the contrary, low income and informal employment can be an obstacle for mHealth utilization and engaging in sustained physical activities.

**Table 1:** Sample characteristics by urban–rural residence (*N* = 1,204).

Variable	Total sample	Urban (*n* = 706)	Rural (*n* = 498)	Test statistic	*p*-value
Residence (%)	100%	58.6%	41.4%	—	—
Age (years), Mean (SD)	38.7 (11.4)	38.2 (11.1)	39.4 (11.8)	t = 1.76	.079
Female (%)	54.2%	53.8%	54.8%	*χ*^2^ = 0.12	.729
Educational attainment (%)				*χ*^2^ = 86.41	<.001
Primary school or less	12.6%	6.9%	20.7%		
Lower secondary school	18.3%	10.5%	29.3%		
Upper secondary school	18.2%	20.3%	15.2%		
Tertiary school	50.9%	62.3%	34.8%		
Household income (million IDR/month) (%)				*χ*^2^ = 92.54	<.001
Below 2.8	26.4%	15.2%	42.3%		
2.8 to 5	31.7%	28.9%	35.7%		
5 to 8	21.5%	24.9%	16.7%		
8 to 12	12.4%	16.5%	6.6%		
More than 12	8.0%	14.5%	2.7%		
Employment stability (%)				*χ*^2^ = 74.63	<.001
Unemployed/irregular work	14.8%	10.6%	20.9%		
Informal employment (unstable income)	29.7%	20.4%	42.8%		
Self-employed/small business	26.9%	28.7%	24.3%		
Formal employment (regular income)	28.6%	40.3%	12.0%		
Smartphone ownership (%)	82.1%	85.0%	78.0%	*χ*^2^ = 9.12	.003
Daily internet access (%)	71.5%	76.8%	64.0%	*χ*^2^ = 18.47	<.001
mHealth utilization, Mean (SD)	3.41 (0.87)	3.63 (0.79)	3.09 (0.88)	t = 9.42	<.001
Sustained physical activity, Mean (SD)	3.38 (0.84)	3.52 (0.81)	3.18 (0.84)	t = 7.21	<.001
BMI classification (%)				*χ*^2^ = 1.82	.001
≥30.0 (obese)	38.5%	36.0%	42.0%		
=25.0–29.9 (overweight)	61.5%	64.0%	58.0%		

Income differences significant at *p* < .001 (exact mean values available upon request). Autonomous -sample t-statistics were for constant variables; *χ*^2^ checks for categorical indicators. Daily internet access was defined as respondents reporting internet use “once a day” or “multiple times a day.” Smartphone ownership and internet access are included to reflect individual-level technological access across urban and rural populations.

Informative statistics (Table 2) specified moderate total engagement in mHealth utilization for tracking physical activity and exercise management. The results indicated higher scores for mHealth utilization among the urban residents than the rural residents. The difference was statistically significant (M = 3.63, STD = .79 vs. M = 3.09, STD = .88; t = 9.42, *p* < .001). The same trend was observed for sustained physical activity, which was higher among the urban residents (M = 3.52, STD = .81) than the rural residents (M = 3.18, STD = .84; *p* < .001). Bivariate correlation examination indicated significant and positive relations between SES and mHealth utilization (*p* < .001, r = .42,), sustained physical activity and mHealth utilization (*p* < .001, r = .48), and sustained physical activity and SES (*p* < .001, r = .36).

**Table 2:** Correlation pattern and informative data.

Variables	Mean	STD	1	2	3
1. Socioeconomic status (SES)	—	—	—		
2. mHealth utilization (MHU)	3.41	.87	.42***	—	
3. Sustained physical activity (SPA)	3.38	.84	.36***	.48***	—

*N* = 1,204. Pearson correlation coefficients reported.

***p < .001.

Pearson's zero-order correlations between variables (here: SES, MHU, SPA) were included in the descriptive statistics to get a preview of the core of the relation among those variables (58). The fact that the correlations represent strong evidence for the conceptual framework may be exaggerated since correlations are less effective in accounting for common variance than the connections between constructs (59). Structural equation modeling (SEM) offers a more suitable technique to simultaneously evaluate these connections. SEM is seen to be more sophisticated than the Pearson correlation coefficient for complex research designs since SEM deivers the second generation multivariate examination that uses factor assessment and path assessment (60). In contrast to the Pearson correlation coefficient, which only assesses the association between two variables, SEM enables the researcher to examine the complexity of inter-variable associations (60).

### 4.2 Measurement framework assessment

#### 4.2.1 Indicator adequacy

The employment of several indicators for each construct (as listed in Supplementary Appendix Table A6 column 2) was also empirically supported. In accordance with the SEM approach, the minimum number of indicators employed for each latent construct should equal three for accurate model identification and estimation of factor loadings (54, 61, 62). The criterion above is satisfied in the present research due to the fact that the number of indicators exceeds three for all constructs involved. Therefore, an ample amount of data on constructs has been obtained to estimate the latter accurately. Through the application of the selected item set, the satisfactory psychometric properties of the scales have been demonstrated (the level of internal consistency proved to be high: composite reliability CR > 0.7, i.e., from .83 to .90; convergent validity has been confirmed by AVE > .50 in both populations: urban and rural) as advised by Hair et al. (57, 63). The indicators utilized in this paper not only make sure comprehensiveness but also avoid any unnecessary complication in the framework formulation procedure. With several indicators being used for every latent variable, it was possible to check whether there existed any discriminant validity among the variables. This was confirmed by the Fornell-Larcker (Table 3) and HTMT tests (Supplementary Appendix Table A2).

**Table 3:** Fornell–larcker matrix by residence types.

Urban (*n* = 706)				Rural (*n* = 498)		
Construct	SES	MHU	SPA	SES	MHU	SPA
Socioeconomic Status (SES)	**.** **783**	.521	.427	.**771**	.513	.420
mHealth utilization (MHU)	.490	.**847**	.562	.482	.**834**	.553
Sustained physical activity (SPA)	.431	.564	.**825**	.424	.555	.**812**

Crosswise values (bold) are AVE's square root. Off-crosswise values are inter-construct links.

#### 4.2.2 Internal consistency

Composite reliabilities (CR) for urban and rural vary between .83 to .90, which is above the recommended threshold of .70, thus establishing good composite reliability (57, 63). To assess the accuracy of the composite reliability (CR), 95% confidence intervals were obtained using a bootstrapping technique with 5,000 iterations. This technique provides a reliable, distribution-free evaluation of variability in reliability coefficients. Table A3 in the Supplementary Appendix provides a confidence interval presentation for bootstrapping CR. All CR values were found to be good for both urban and rural cohorts. The most noteworthy aspect is that the 95% confidence intervals, which were obtained through bootstrapping, were narrow and well above the suggested minimum level of .70. As an example, mHealth utilization demonstrated CR of .90 [95% CI = (.88, .92)] among urban responders and .87 [95% CI = (.84, .89)] among rural responders. These outcomes reflect robust internal consistency and stable reliability estimates with minimal variation across samples. Finally, AVE (average variance extracted) between .60 to .69, which outdo the minimum required .50, thus establishing good convergent validity (57, 63). It is clear that the constructs of SES, mHealth utilization, and sustained physical activity have good reliability and validity for both groups.

#### 4.2.3 Indicator reliability

All items have been assessed based upon the standardized factor loadings (*λ*) of each indicator regarding the latent variable using the method of CFA within SEM. Those items having standardized factor loadings ≥.70 are deemed acceptable since they denote the significance of the contribution made by the indicator towards the latent variable (64). From Table 4, it is clear that the indicators for all the three latent variables, i.e., SES, mHealth utilization (MHU), and sustained physical activity (SPA), are in accordance with the criterion set in urban and rural samples. For instance, the factor loading for MHU1 in the urban cohort was .88 (*λ* = .88), whereas the factor loading for SPA1 in the urban cohort was .83 (*λ* = .83), which denotes robust convergent validity within constructs. Though SES3 and MHU5 show lower loadings than most other items (respectively, *λ* = .74 and *λ* = .77), their values exceed the critical level as well. The inclusion of SES3 and MHU5 can be considered legitimate because theoretically, they exhibit important dimensions of their respective constructs. Empirically, their inclusion has a positive influence on the values of CR and AVE (CR > .80, AVE > .50). Hence, all the indicators were kept because their presence did not affect the validity of measurement.

**Table 4:** Measurement framework outcomes.

Construct	Indicator	Urban (*n* = 706) *λ*	AVE	CR	*α*	Rural (*n* = 498) λ	AVE	CR	*α*
Socioeconomic status (SES)	Education level	.78***	.60	.86	.82	.76***	.60	.83	.79
	Household income	.82***				.79***			
	Employment stability	.74***				.71***			
mHealth utilization (MHU)	MHU1	.88***	.69	.90	.88	.86***	.66	.87	.85
	MHU2	.85***				.82***			
	MHU3	.81***				.79***			
	MHU4	.79***				.81***			
	MHU5	.77***				.74***			
	MHU6	.86***				.83***			
Sustained physical activity (SPA)	SPA1	.83***	.64	.88	.85	.81***	.63	.85	.82
	SPA2	.80***				.76***			
	SPA3	.79***				.74***			
	SPA4	.81***				.81***			
	SPA5	.77***				.77***			
	SPA6	.75***				.75***			

*λ*, item loading; *α*, Cronbach's alpha.

*** *p* < .001.

#### 4.2.4 Construct-level results

Table 4 exhibits the measurement framework outcomes attained for Urban (*n* = 706) and Rural (*n* = 498) groups. The entire item loadings for both clusters have robustness and statistical significance, with numerical representatives between .71 to .88, *p* < .001, designating satisfactory indicators’ consistencies. The analysis using SEM showed that the concept of SES had three constructs, which included education level (SES1), household income (SES2), and employment stability (SES3). There were no preassigned weights used in creating the composite index for this construct; the weights for individual constructs were based on item loadings obtained through the process of confirmatory factor analysis (CFA). The factor loading of SES1, SES2 and SES3 were relatively high (*p* < .001) in rural and urban samples (see Table 4). Household income had the highest factor loading values, which were 0.82 and 0.79 in the urban and rural cohorts, respectively. Employment stability had the lowest factor loadings (urban *λ* = .74; rural *λ* = .71). Consistency in the measurement of SES across the two cohorts is specified by the similarity of loadings across them. While the loadings of SES2 (education) is relatively high (urban *λ* = .78; rural *λ* = .76), this construct can be viewed as a combination of all three measures rather than a unique measure. To prove that the findings are not the effect of only one measure, sensitivity examination was done through framework's appraisal with the use of distinct measures (SES1, SES2 and SES3 separately). While one measure (SES2) was substantial when used separately, it is the three-measure combination of SES that produced best model fitness (CFI=.948; RMSEA=.042) with this three-measure amalgamation, stable relationships with mHealth utilization and sustained physical activity were maintained, corroborating the outcomes’ robustness.

#### 4.2.5 Model fitness

Goodness-of-fit indicators have been considered for determining the measurement framework's goodness-of-fit (view Supplementary Appendix Table A5). The fitness of the CFI and TLI measures being more than .90 indicates that the measurement model fits well when compared with the null model. The RMSEA measure having a value less than .05 suggests that there is an excellent fit between the proposed framework and the population's covariance matrix. The SRMR measure, having a value less than .05, indicates that the differences between the observed and implied covariance matrices are small. In particular, it can be stated that this model is quite a well-fitted one in rural and urban samples, which ensures the validity of this construct model.

#### 4.2.6 Discriminant validity

Discriminant validity was confirmed with Fornell-Larcker principle (Table 3). It was found that the matrices for rural and urban groups were psychometrically sound for structural examination as the crosswise numerical representatives outstrip inter-construct links. In addition to the Fornell-Larcker test, discriminatory validity was also gauged using heterotrait-monotrait ratio (HTMT) (64). According to the results for the rural and urban samples, the HTMT ratio was less than .85, and all pairs of constructs passed the requirement (urban dataset: .42–.56; rural dataset: .42–.55), which is considered to be a very high measure of discriminatory validity (see Supplementary Appendix Table A2). The obtained HTMT ratio was .68 between mHealth utilization and sustained physical activity by the urban cohort, which is still within acceptable range. This finding helps further validate the empirical distinctness of the indicators, alleviating concerns about the potential leniency of the Fornell-Larcker standard.

Cross-loadings were applied to determine if the indicators were significantly loaded on some other construct. The cross-loadings test was performed using the standardized loadings on all the latent variables. It was mandatory for each indicator that its loading on the relevant factor should be more than its loading on any other factor. In the present study, the cross-loadings of the entire items of SES, SPA, and MHU constructs are exhibited in the Supplementary Appendix Table A6. Each item loads most on its own construct, which is displayed in bold numbers. For instance, the entire items gauging MHU load more on MHU (0.85–0.89); SPA loads more on SPA (0.83–0.89); SES loads more on SES (0.84–0.87). Here, the discrepancy between the loading on the primary factor and the maximum cross-loading among all the variables was greater than .20, which reflected discriminant validity (64). There was a consistency in these patterns within rural and urban samples, which verifies that every indicator exclusively contributed to its assigned construct.

#### 4.2.7 Measurement invariance results

To test the validity of structural paths involving individuals from urban areas and rural areas, measurement invariance was carried out according to standard practices followed in SEM. To determine the similarities of factor structure (number of factors and pattern of factor loadings) among the two cohorts, configural invariance was conducted (65). Based on the results, there is equal overall factor structure of SES, MHU, and SPA measures among urban (*n* = 706) and rural (*n* = 498) samples. Besides, metric invariance was conducted to examine whether there is any equality of factor loadings across the two cohorts (66). Chi-square differences test results revealed that there is no difference in the factor loadings (ΔCFI < .01). Thus, it can be inferred that the items have similar contributions to their latent constructs in both cohorts. Further, in case scalar invariance was achieved, equality of item intercepts was examined (66). Minor variations were noted, although these had no impact on the associations within the structural model, which allows validity in comparing latent means with path coefficients.

The test for measurement invariance between two cohorts from an urban-rural sample was conducted in sequence (see Supplementary Appendix Table A1). First, the baseline framework exhibited excellent fitness (CFI = .952; RMSEA = .041) which indicates that the same factor structure was found to exist in the two samples. Second, while conducting measurement invariance test regarding factor loading, some discrepancies were observed in the value of CFI (.003) indicating that metric invariance existed. Full scalar invariance did not exist but partial scalar invariance did exist because only a few item intercepts were free (*Δ*CFI = .002). It therefore means that the constructs under consideration have been measured equally in the two sample, which justifies the assessment of path estimates in the multi-group SEM.

### 4.3 Structural framework assessment

The hypothesized framework possesses an excellent fitness with the data, SRMR = .045, RMSEA = .051, TLI = .948, CFI = .957, *p* < .001, *χ*^2^ (df = 168) = 438.15. Table 5 exhibits that SES had a positive significant links with mHealth utilization (*p* < .001, *β* = .44) and sustained physical activity (*p* < .001, *β* = .21). It was unearthed that mHealth utilization had a significant linkage with sustained physical activity (*p* < .001, *β* = .46). Five thousand samples in bootstrapping examination divulged that SES had a substantial indirect linkage to sustained physical activities, controlling for mHealth utilization [*β* = .20, 95% CI (.16, .24)]. The empirical outcomes imply that all significant relations in the research have been validated through SEM. From the examination, it can be observed that under the condition where all other latent variables are held constant, the structural frameworks have statistical significance, such as SES → MHU (*β* = .44, *p* < .001); MHU → SPA (*β* = .46, *p* < .001), and SES → SPA (*β* = .21, *p* < .001). Hence, it can be deduced that the relations are robust within a multivariate context and cannot be observed at the zero-order level alone (59, 60).

**Table 5:** Structural framework outcomes.

Hypothesized path	*β*	SE	95% CI	*p*-value	Outcome
Direct associations					
H1. SES → MHU	.44	.04	[.36, .52]	<.001	Supported
H2. MHU → SPA	.46	.04	[.38, .54]	<.001	Supported
H3. SES → SPA	.21	.05	[.12, .30]	<.001	Supported
Indirect association					
H4. SES → MHU → SPA	.20	.02	[.16, .24]	<.001	Supported

*N* = 1,204. Standardized coefficients (*β*) reported. Confidence intervals (CI) derived from 5,000 bootstrap samples; SPA, Sustained physical activity; MHU, mHealth utilization; SES, Socioeconomic status.

As depicted in Table 5, the effect coefficients are moderate-to-strong for the link between SES and mHealth utilization (SES → MHU) was *β* = .44, while for the link between mHealth utilization and sustained physical activity (MHU → SPA) was *β* = .46. effect coefficient for the link between SES and sustained physical activity (SES → SPA) was *β* = .21, and this is relatively small compared to the other two relations. On the other hand, the indirect effect was *β* = .20, which is moderate and comparable in magnitude to the direct link. This suggests that mHealth utilization exemplifies a consequential pathway connecting SES with SPA.

Even though this work's primary aim was to compare behaviors in urban vs. rural areas, the survey obtained information about responders’ age and gender. To explore whether there is a potential moderating role of age and gender in regard to the association of SES, MHU and SPA variables, further SEM analyses were performed using age groups and gender subgroups as moderators. The findings revealed no significant differences among the structural paths (SES → mHealth → SPA), regardless of the gender and age group (by Δ*χ*^2^ test) (*p* > .05). This indicates that the interaction effect between the variables remains the same regardless of age group and gender. Moreover, it suggests that the urban-rural variations will most likely remain unaffected by these demographic characteristics. Thus, the rural-urban difference is mainly due to dissimilarities in setting such as digital access, infrastructure, and socio-economic factors. It clearly indicates that the SEM outcomes is generalizable for all adult Indonesians.

### 4.4 Multi-group SEM for urban–rural disparities

To assess spatial differences, a multi-group SEM examination was used to compare subsamples of urban and rural residents. Configural invariance tests disclosed excellent fit for the overall framework (SRMR = .048; RMSEA = .052; CFI = .955). Metrical and scalar invariance tests reinforced the framework (ΔCFI = .004 and ΔCFI = .006, respectively); thus, structural contrasts could be proceeded. Table 6 displays that the link between SES and mHealth utilization was stronger in the urban than the rural group (*p* < .001, *β* = .49 vs. *β* = .34); group differences had statistical significance (Δ*χ*^2^ = 9.87, *p* = .002). The mHealth utilization had a significant positive relation with sustained physical activity for both clusters but was stronger for the urban than the rural group (*β* = .52 vs. .37, Δ*χ*^2^ = 11.24, *p* = .001). The SES → SPA path was significant for the urban group (*p* < .001, *β* = .24) and weaker within the rural (*p* = .041, *β* = .12); marginal group differences emerged (Δ*χ*^2^ = 3.84, *p* = .050).

**Table 6:** Multi-group SEM comparison of structural paths: urban vs. rural.

Structural path	Rural (*n* = 498) *β*	Urban (*n* = 706) *β*	*Δχ*^2^ (1 df)	*p*-value (difference test)	Interpretation
H1. SES → MHU	.34**	.49**	9.87	.002	Stronger in urban
H2. MHU → SPA	.37**	.52**	11.24	.001	Stronger in urban
H3. SES → SPA	.12*	.24*	3.84	.050	Marginally stronger in urban

Indirect path	Rural β (95% CI)	Urban β (95% CI)		Interpretation	
H4. SES → MHU → SPA	.13 [.09, .17]***	.25 [.20, .30]***		Stronger mediation in urban	

Endogenous variable	Rural R^2^	Urban R^2^		Interpretation	
MHU	.12	.24		Higher in urban	
SPA	.21	.39		Higher in urban	

Normalized coefficients (*β*).

Δ*χ*^2^ reflects chi-square difference test comparing constrained and unconstrained frameworks. SPA, Sustained physical activity; SES, Socioeconomic status. *R*^2^ denotes the ratio of variance explained in endogenous variables.

* *p* < .01.

** *p* < .05.

*** *p* < .001.

Indirect associations via mHealth utilization appeared stronger in urban than in rural settings (*β* = .25, 51% mediated vs. *β* = .13, 42% mediated). The unconstrained framework had excellent fit (CFI = .951; RMSEA = .053). When the paths were constrained, the framework had poor fit (Δ*χ*^2^ = 21.36, *p* < .001), which confirmed the existence of structural heterogeneity. The R^2^ for the explained variance was observed to be higher for the urban compared to the rural context for sustained physical activity and mHealth utilization (Table 6).

From the multi-group SEM test outcomes exposed in Table 6, it appears that structural relations are more significant among urban cohort compared to those found in rural samples. Chi-square differences were employed to assess differences in group significance, such as Δ*χ*^2^ = 9.87, *p* = .002 for SES → MHU paths difference and Δ*χ*^2^ = 11.24, *p* = .001 for MHU → SPA differences. The attainment of partial scalar invariance makes sure that the differences identified are real disparities within structural relations and not the result of measurement mistakes. From the assessment outcomes, it is clear that despite the theoretical model being applicable to rural and urban societies, there are disparities in relation to their degree of relations, and hence there will also be disparities in relation to mHealth utilization and SPA by the two sample categories.

The outcomes of multi-group SEM assessment in Table 6 explicitly compared rural (*n* = 498) and urban (*n* = 706) samples. Associations between structures have been shown to be higher in an urban environment because individuals with a greater SES status tend to use mHealth more frequently, which subsequently enhance SPA. While findings in rural samples also reflect the existence of associations, lower scores may point to the existence of context-related variables like discrepancies in accessibility to health technologies, digital infrastructures, or other environmental limitations that affect mHealth utilization. Besides, the finding about SES-SPA association mediated by mHealth utilization is appreciably greater intensity within urban regions than rural zones.

Concerning the configural and metric invariance analyses, the difference in the path coefficient between SES and MHU (i.e., SES → MHU) as well as MHU and SPA (i.e., MHU → SPA) may indicate the existence of a genuine difference between the cohorts which does not result from the violation of equivalence in measurements. Hence, statistical meaningfulness and robustness of inter-variable associations were found to be stronger in urban areas (e.g., SES → MHU: Δ*β* = .15, *p* = .002; MHU → SPA: Δ*β* = .15, *p* = .001).

Further, as displayed in Table 7, the RQs are answered: (1) SES has a positive link with mHealth utilization, (2) SES and mHealth utilization positively relate to sustained physical activities, (3) mHealth utilization has a partial mediation between SES and sustained physical activities, and (4) the outcomes of multi-group SEM designate that the structural relation is significantly different between the urban and rural samples, and the relations are stronger in urban.

**Table 7:** Alignment between RQs, hypotheses, and empirical outcomes.

Research question		Corresponding hypothesis	Path	Empirical answer
RQ1	How are SES, mHealth utilization, and sustained physical activity related among overweight and obese adults?	H1, H2, H3	SES → MHU	Supported
			MHU → SPA	
			SES → SPA	
RQ2	Does mHealth utilization mediate the association between SES and sustained physical activity?	H4	SES → MHU → SPA	Supported
RQ3	Do the indirect associations among SES, mHealth utilization, and sustained physical activity differ between urban and rural cohorts?	H5	Multi-group SEM contrast	Supported. (Consistently stronger relations in urban)

## 5 Discussion and implications

### 5.1 Discussion of results

The first experiential outcomes suggest that a positive relation between SES and mHealth utilization esist. This result is in line with Koivusilta et al. (23), indicating that the accessibility to smartphones and internet connection, affordability of data services, as well as literacy in mHealth apps require people to have financial and cognitive resources. People who have more educational, economic, and work stability benefits tend to utilize more mHealth apps in maintaining physical activities, consistent with Aljerf et al. (67). Nevertheless, the finding is against that of Chang et al. (68), who discovered that SES is not linked to mHealth utilization. This contradiction might be due to contextual factors because the development of mHealth services in the USA is facilitated through insurance and public health, while in Indonesian contexts, mHealth utilization might be dependent on SES.

The mechanisms underlying the observed positive correlation between SES and mHealth utilization include: (a) access to technologies and connectivity, (b) health literacy and cognitive abilities, (c) motivation and lifestyle factors, and (d) relation within SEM framework. First, persons from high SES have easier access to mobile devices and internet connection that allows them to make use of mHealth applications (6, 23). In Indonesia, the problem gets even worse due to different development levels in urban and rural zones (i.e., lack of 3G/4G connection or access to smartphones). Second, high SES can be related to high levels of health literacy that include capability of understanding, interpreting, and acting upon health information online (6, 23). Besides, familiarity with online health information and high cognitive abilities can improve effectiveness in utilizing mHeath apps. Third, people with higher SES could have enough free time for self-care activities concerning their health and physical condition, which is aligned with involvement in planning and tracking physical exercises using mHealth apps (6, 7). This disparity can contribute to the relation of SES with mHealth utilization. Fourth, although the bivariate correlation coefficient is the first step in analyzing statistical data, in the overall SEM model, there are numerous other variables, including SES, that are considered, and finally, it is revealed that SES still strongly influences mHealth utilization (*β* = .44, *p* < .001). In other words, in addition to technological usage, health literacy and lifestyle also play their roles.

The second findings established that mHealth utilization was linked to sustained physical activity, which was more significant for the urban sample than rural. This is a contrast to Golbus et al. (27)'s, which pinpointed that no significant improvement for patients who utilized mHealth in six months. This could be due to the fact that in their research, the clinical context might be limiting the benefits of digital health, while for this paper, the self-directed mHealth utilization might be vital for self-managed exercise. However, the findings support Zhang et al. (12)'s work, which identified digital health's benefits, even for rural sample who had lower connectivity. Multi-group SEM makes it possible to separate the link of SES and the individual and rural-urban differences as SES can be treated as a latent variable, including three components, i.e., education, income, and employment stability. However, there might be an unobservable confounder in the form of the quality of infrastructure and labor market conditions. In particular, rural responders who have physical activity connected with their jobs will score lower for SPA if it was non-recreational and measured using digital devices. Likewise, mHealth utilization could remain limited by infrastructure obstacles that extend outside individual SES.

The third outcomes exhibit that SES directly relates to sustained physical activity, contrasting with an earlier discovery (32), who found no association between education and physical activity. A contextual difference may explain this discrepancy. In their cohort, higher wealth was linked to inactivity, possibly due to motorized transport and sedentary lifestyles. In contrast, this paper's finding suggest higher SES supports sustained activity through access to facilities, supportive networks, and health resources.

The mediation inquiry revealed that mHealth utilization partially mediates the link between SES and sustained physical activity. People with more economic and education advantages tend to have more opportunities to use mHealth, which is associated with the persistence of existing advantages. The stronger mediation relation was disclosed in urban areas, which underlines digital inequalities, with more urban resources enhancing the behavioural returns for digital engagement, as per AlJerf et al. (67). It reinforces the work of Rick et al. (33), which suggested a notable mediating role of mHealth utilization among urban workers. The research has further added value by exploring the general citizen with an obesity/overweight issue, as opposed to just workers, and revealed a stronger mediation urban-rural linkage.

Lastly, SES was uncovered to have stronger link with mHealth utilization in the urban area. This is because the facilities are better and exposure is higher. This outcome reinforces an earlier finding which also specified that the linkage between mHealth utilization and sustained physical activity was stronger in urban area, i.e., Aljerf & Aljerf (69). The constraints in rural area, like the lack of facilities, work-related issues, and connectivity, are likely to minimize the returns (69). More specifically, urban areas and rural areas would differ on grounds of availability of parks, gymnasiums, sites for walking, and mode of transport used by citizens, all of which may encourage or discourage exercise. In an urban setting characterized by many places for doing physical exercises, such as gymnasiums, cycle tracks, and sports clubs, there is high probability that people will do physical exercises in their free time. There is high probability of individuals belonging to a rural background being involved in physical exercises while performing occupational duties. Thus, they have less tendency to be captured by mHealth tracking tools. Such environmental disparity may partially misperceive the detected relations among SES, mHealth utilization, and sustained physical activity. Besides, there are social and cultural influences. Social influence consists of certain individuals like families, friends, and other types of social groups that are present within a society along with the society's culture, which influences how people participate in exercising and mHealth utilization. The societal environment of people who live in urban areas promotes the usage of mHealth apps for exercising. On the other hand, people living in rural societies would prefer traditional means in making healthier decisions about their lifestyles.

Infrastructure and labor context can be influential confounders for mHealth utilization (MHU) and sustained physical activity (SPA) within urban and rural cohorts. This issue was solved through inclusion of mobile phone access and Internet usage by the respondents individually. Smartphone ownership was assessed using a binary self-report item (“Do you currently own a smartphone?”). Internet access was measured using a frequency-based item, which was subsequently dichotomized to indicate daily access (use of internet “once a day” or “multiple times a day” (see Supplementary Appendix Questionnaire Part 4). From the descriptive statistics, 78% of those living in the rural areas owned mobile phones, whereas 64% of the same population accessed the Internet each day (see Table 1). However, after including such factors into the SEM assessment, only minimal reductions of association coefficients (for SES → MHU → SPA) were seen, although significance remained intact, which means access constraints are not sufficient to statistically explain the observed associations.

### 5.2 Implications for theory and policy

#### 5.2.1 Implications for theory

This paper's findings have some theoretical implications. It contributes to digital health inequality, structural modeling in public health, and obesity management research. It reconceptualizes mHealth utilization as both an independent and mediating variable rather than solely a dependent outcome. Earlier publications primarily assessed mHealth utilization as shaped by socioeconomic and demographic [e.g., (8, 17, 70)], improving understanding of access and acceptance but offering limited insight into how mHealth apps translate structural resources into health behaviors. By modeling mHealth utilization as predictor and mediator, this paper advances a socio-technical perspective in which mHealth technologies link SES to sustained physical activity. The findings display that mHealth utilization is shaped by SES while also influencing behavioral outcomes and partially mediating inequality, extending prior work that focused mainly on determinants of mHealth utilization (12, 20). Besides, mHealth utilization's mediation nature differs among urban and rural groups, as reflected in the multi-group SEM outcomes. As earlier works on the mHealth-obesity linkage is predominantly conceptual, e.g., (16, 71), the current paper makes a contribution to extant publications via empirical provision concerning mHealth's function as a management tool for obesity.

The findings of this research illustrate the substantial associations between SES on mHealth utilization and sustained physical activities. The finding also illustrates how the role of mHealth mediates the SES-mHealth behaviors relation. Besides, the multi-group SEM approach indicated that the degree of associations between SES, mHealth utilization and SPA is more intense in urban samples than rural community members, confirming that behavioral and digital divides exist. There are significant contributions that this research adds to the current literature regarding SES and health behaviors. Firstly, the research provides an empirical demonstration on how socioeconomic disparities create gaps in digital health technologies, thus creating digital inequalities on SPA. Secondly, there are health disparities in Indonesia between urban and rural areas based on SES that affects mHealth utilization and SPA. Third, the research papers provide empirical findings on partial mediation, with an emphasis on mHealth utilization for reducing disparities concerning health behaviors.

#### 5.2.2 Implications for policy

The implications of the policy need to be taken into consideration carefully because the empirical outcomes were based on a cross-sectional report. Based on Table 6, a higher association between SES, mHealth utilization, and sustained physical activity was observed in urban communities as compared to rural areas. Structural examination revealed the presence of higher mediation of mHealth utilization among the urban community cohort. The paper's outcomes suggest that contextual factors, rather than effectiveness of interventions, may explain these linkages. From the perspective of policy making, it may be noticed that efforts have to be made to improve enabling factors (e.g., digital accessibility, affordability, and literacy) in the rural environment, while in the urban environment, it is necessary to leverage extant infrastructure. One important thing which needs to be considered is that no causal or policy impact can be established on the basis of the results obtained. Disparities are instead highlighted by the findings, and further longitudinal and experimental evaluation is warranted before interventions are scaled. However, the empirical outcomes are indicative of the importance of considering digital divide and urban-rural gap issues in Indonesia. Policy implications may include formulation of: (a) Interventions aimed at increasing access to mHealth technology among rural communities; and (b) Programs promoting digital literacy to potentially reinforce mHealth benefits on sustained physical activities for obesity control.

In the context of Indonesia, there are a number of suggestions that emerge from the outcomes of this work. Firstly, policymakers may consider creating programs that promote digital health, where they should emphasize using different digital devices by improving infrastructure, providing affordable mobile phones for people, and designing mobile health apps that are easily understandable by people living in rural and low-SES areas. So far, apps like *Alodokter* and *Mobile JKN* are widely used across Indonesia to chat with doctors for appointment booking and reading healthcare articles in Indonesian language using local context and helps low literacy; however, most apps in Indonesia are urban-first designs. Indonesia does also have fitness apps for supporting sustained physical activities (like *Fita* and *Fumai*) but most are not truly designed for low-SES rural users as barriers may still include internet access, digital literacy, and complex interfaces. Secondly, integrated public health programs could incorporate financial incentives for people with low SES levels and mobile apps aimed at performing physical exercises. Thirdly, the challenges that arise when making the digitization of the *Posyandu* (a community-based health service in Indonesia that operates at village level and is a main part of primary healthcare) possibly due to the absence of necessary technical skills among healthcare workers operating at the grassroots level. Hence, it could be reasonable to address the problem of digital illiteracy of people who live in rural and low-SES areas because this could be an effective way of resolving digital inequality problems. It may be equally important to adopt an evaluation framework that quantifies the utilization of mHealth technology and physical activity levels across different socioeconomic strata.

At a wider scope, despite the situational nature of the research, the conclusions reached by this work indicate that the mechanisms are potentially capable of being applied for generalizing from a theoretical point of view. Such a mechanism can be applied to developing countries suffering from digital and rural divides; therefore, identical conclusions would be made regarding the influence of SES on mHealth utilization and subsequent behavior changes. Moreover, the problems inherent in LMICs such as resource shortages, health literacy, and infrastructure problems can suggest real-world applicability of this paper's theoretical approach. Nevertheless, the magnitude of these associations and comparative significance of SES, cultural and infrastructure aspects will be reliant on the context and need local validation and adaptation. Hence, other policymakers of LMICs could consider several valuable lessons proposed by the paper to formulate relevant policies. Some of these include improving digital technology in the countryside, developing mHealth apps that can be used even in areas with less smartphone access, and following a community-based strategy to supplement mHealth programs along with encouraging physical exercise.

## 6 Conclusion, limitations and upcoming works

### 6.1 Conclusion

The paper's findings unveil that mHealth utilization's function in obesity control in Indonesia varies according to socioeconomic inequalities and rural-urban disparities. The outcomes disclose that mHealth utilization partially mediates the link between SES and sustained physical activity, yet this link does not completely eliminate inequalities. Moreover, rural-urban disparities significantly moderated some of the main pathways, which implies that digital health inequalities might not be evenly distributed across space. While mHealth utilization might hold promise for obesity control, its usefulness relies on larger societal and infrastructural circumstances in which it is implemented. If not implemented from an equity perspective, the expansion of digital health technology might even contribute to higher inequalities rather than alleviate them. Amalgamating technological innovations with structural investments to certify inclusive and equitable health outcomes.is hence a must for policymakers.

### 6.2 Limitations and upcoming works

However, several weaknesses exist. Firstly, the data employed in the current paper is cross-sectional data, which only offer information for a particular span of time. Hence, it should be noted that the cause-and-effect relation proposed by SEM needs to be considered as statistical association. Although SES and mHealth utilization are considered as reliable predictors of sustained physical activities, there exists the possibility of reverse causality between the two. Longitudinal researches should be done in the future to determine the influence of mHealth utilization and SES on sustained physical activities. Secondly, the recall bias may influence the assessment of sustained physical activity and mHealth utilization, as they were based on self-reports. For future research, the use of objective measures, such as GPS coordinates and data from wearables and mHealth applications, would contribute to improved validity and reliability of results. Examples of objective measures that can be used in upcoming works are: (a) Exercise via the use of pedometers/accelerometers, (b) Body mass index via direct anthropometric measurements, and (c) mHealth utilization via app usage logs. The use of these measures will improve the accuracy of data collection, especially when the individuals to be studied differ in terms of occupation and technology adoption (e.g., rural vs. urban).

Third, other mediators like digital literacy, motivational variables, and built environment variables are not included in the framework. While this paper used SEM that adjusts for the effect of SES, it is essential to note that there were no variables concerning socio-environmental context. By excluding variables from the study as a measure to control confounders, it limits the ability to discern the effects of SES and urban/rural status on each other. Thus, any differences that may exist are caused by several elements such as socioeconomic, environmental, cultural, and infrastructural aspects. Upcoming works can employ variables like infrastructure quality and geographic spaces (e.g., parks and transportation availability) and social network variables along with health care access and connectivity as objective measures to have more comprehensions regarding the difference between behavioral and structural determinants of mHealth utilization. Incorporating such variables into the model, upcoming works can estimate the effect of these variables on associations through multi-group SEM and hierarchical modelling. Besides, because this work does not have area-level infrastructure data, causation cannot be established in the link between SPA and the dependent variable. Multilevel studies are warranted in future research.

Furthermore, cross-country findings must be attained to assess the bearing of national differences in digital infrastructure on inequality. The need for replication of this work in other nations so that the external validity of the research can be determined is important. Researchers should conduct comparative studies among various nations while considering the differences in their infrastructural, cultural, and working environments. This will give better understanding about the association between SES and technological use in relation to health behavior within different perspectives.

Moreover, using SES as a latent construct that can be measured through various indicators allows for the opportunity to take into account the complexities involved in the definition of SES without limiting to a particular measure of SES. Hence, the SEM model can account for the variance of each component, which includes education, income, and occupational stability. It reduces the possibility that the empirical outcomes are influenced by just one aspect of SES like education. While the distinction between urban and rural may provide good differentiation regarding socio-environmental conditions, it is not enough for the whole Indonesian perspective. Hence, subsequent researches should apply a more appropriate technique in analyzing their data, either through the application of a multilevel investigation approach or through stratifying the data based on the behavior differences noted between peri-urban areas, small towns, and rural settings. In relation to contextual variables, possible factors could include the regional infrastructure index or labor dynamics.

## Data Availability

The original contributions presented in the study are included in the article/Supplementary Material, further inquiries can be directed to the corresponding author/s.
