# Supplementary material for: Beyond the digital divide: multi-group SEM examination of socioeconomic status, mHealth utilization, and urban-rural physical activity disparities in Indonesia
Source: Front Digit Health. 2026 May 29;8:1831481. doi: 10.3389/fdgth.2026.1831481 (PMC13260068; doi:10.3389/fdgth.2026.1831481)
Supplement: Supplementary file 1 [file Supplementaryfile1.docx]

# **Appendix**

## **Questionnaire**

### ***Part 1: mHealth utilization (MHU)***

Please specify how much you approve of every declaration.
1 = Fully disapprove | 2 = Disapprove | 3 = Neutral | 4 = Approve | 5 = Fully approve

| Code | Indicator/item | 1 | 2 | 3 | 4 | 5 |
| --- | --- | --- | --- | --- | --- | --- |
| MHU1 | Using mHealth applications motivates me to maintain regular physical activity. | ☐ | ☐ | ☐ | ☐ | ☐ |
| MHU2 | Using mHealth applications for managing my body weight and physical activity is a pleasant experience. | ☐ | ☐ | ☐ | ☐ | ☐ |
| MHU3 | Using mHealth applications helps me monitor and manage my physical activity more effectively. | ☐ | ☐ | ☐ | ☐ | ☐ |
| MHU4 | I regularly spend time using mHealth applications to track my physical activity or body weight. | ☐ | ☐ | ☐ | ☐ | ☐ |
| MHU5 | Using mHealth applications allows me to access my health and physical activity information quickly. | ☐ | ☐ | ☐ | ☐ | ☐ |
| MHU6 | I frequently use mHealth applications to support my physical activity or weight control. | ☐ | ☐ | ☐ | ☐ | ☐ |

### ***Part 2: Sustained physical activity (SPA)***

Please specify how much you approve of every declaration.
1 = Fully disapprove | 2 = Disapprove | 3 = Neutral | 4 = Approve | 5 = Fully approve

| Code | Indicator/item | 1 | 2 | 3 | 4 | 5 |
| --- | --- | --- | --- | --- | --- | --- |
| SPA1 | I engage in physical activity regularly every week. | ☐ | ☐ | ☐ | ☐ | ☐ |
| SPA2 | I maintain a consistent routine of physical activity. | ☐ | ☐ | ☐ | ☐ | ☐ |
| SPA3 | I allocate sufficient time for physical activity in my daily or weekly schedule. | ☐ | ☐ | ☐ | ☐ | ☐ |
| SPA4 | I continue to perform physical activities even when I am busy. | ☐ | ☐ | ☐ | ☐ | ☐ |
| SPA5 | I try to maintain physical activity as part of my lifestyle. | ☐ | ☐ | ☐ | ☐ | ☐ |

**SPA6. Please specify the degree of your physical activities during leisure period (plus transport like cycling or walking to workplace) during past year.**
**Select one answer only:**

☐ 1. Almost inactive – Nearly fully inactive or simple physical activities below 2 hours weekly (like reading, watching TV, sitting activities).
☐ 2. Lightly active – Simple physical activities around 2–4 hours weekly (like walking, cycling, simple workout, simple gardening).
☐ 3. Moderately active – Simple physical activities of above 4 hours weekly or moderate-to-heavy activities of 2–4 hours weekly (activities that make you sweat or breathe harder).
☐ 4. Highly active – Strenuous physical activities of above 4 hours weekly or consistent intensive workouts more than once weekly.

### ***Part 3: Socioeconomic status (SES)***

Please choose one response only for every query.

**SES1. The uppermost degree of formal schooling I have accomplished is…..**
☐ 1. Primary school or less
☐ 2. Lower secondary school
☐ 3. Upper secondary school
☐ 4. Tertiary school (Diploma, Bachelor’s degree and above)

**SES2. What is your average monthly household income (in million IDR)?**
☐ 1. Below 2.8
☐ 2. 2.8 to 5
☐ 3. 5 to 8
☐ 4. 8 to 12
☐ 5. More than 12

**SES3. My present-day employment circumstance is…..**
☐ 1. Unemployed or irregular work
☐ 2. Informal employment with unstable income
☐ 3. Self-employed or small business with moderately stable income
☐ 4. Formal employment with regular income

### ***Part 4: Demographic, health, and digital behavior information***

Please answer all questions.

**D1. My age:** ______ years old

**D2. My gender:**
☐ 1. Woman
☐ 0. Man

**D3. I am living in:**
☐ 1. Urban (town/city)
☐ 0. Rural (countryside/village)

**D4. What is your height and body weight?**
Weight: ______ kg
Height: ______ cm

**D5. How often do you access the internet (via any device)?**
☐ 0. Never
☐ 0. Less than once a week
☐ 0. Several times a week
☐ 1. Once a day
☐ 1. Multiple times a day

**D6. Do you currently own a smartphone?**
☐ 1. Yes
☐ 0. No

## **Table A1. Measurement invariance testing among urban and rural cohorts**

| Model | RMSEA | TLI | CFI | df | χ² | ΔCFI | Interpretation |
| --- | --- | --- | --- | --- | --- | --- | --- |
| Configural invariance | .041 | .944 | .952 | 348 | 812.45 | — | Acceptable fit; same factor structure |
| Metric invariance | .040 | .945 | .949 | 366 | 835.62 | .003 | Supported (ΔCFI ≤ .01) |
| Scalar invariance | .042 | .942 | .944 | 384 | 878.91 | .005 | Partially supported |
| Partial scalar invariance | .041 | .944 | .947 | 380 | 856.37 | .002 | Supported |

**Notes:** ΔCFI ≤ .01 indicates invariance. Partial scalar invariance achieved by freeing selected item intercepts.

## **Table A2. HTMT for discriminant validity**

| Constructs | **Rural (n = 498)** | | | **Urban (n = 706)** | | |
| --- | --- | --- | --- | --- | --- | --- |
|  | SES | MHU | SPA | SES | MHU | SPA |
| SES | — |  |  | — |  |  |
| MHU | .59 | — |  | .61 | — |  |
| SPA | .50 | .66 | — | .52 | .68 | — |

**Notes:** HTMT values < .85 indicate adequate discriminant validity (conservative criterion).

## **Table A3. Composite reliability with bootstrapped 95% confidence intervals**

| Construct | Urban CR | 95% CI | Rural CR | 95% CI |
| --- | --- | --- | --- | --- |
| Socioeconomic status (SES) | .86 | [.83, .89] | .83 | [.80, .86] |
| mHealth utilization (MHU) | .90 | [.88, .92] | .87 | [.84, .89] |
| Sustained physical activity (SPA) | .88 | [.85, .90] | .85 | [.82, .88] |

**Notes:** Confidence intervals derived from 5,000 bootstrap samples. All CR values exceed the recommended threshold of .7.

## **Table A4. Assessment of common method bias using a common latent factor**

| Construct | Indicator | Standardized loading (no CLF) | Standardized loading (with CLF) | Δ Loading |
| --- | --- | --- | --- | --- |
| SES | Education | .78 | .76 | -.02 |
| SES | Income | .82 | .80 | -.02 |
| SES | Employment | .74 | .72 | -.02 |
| MHU | MHU1 | .88 | .86 | -.02 |
| MHU | MHU2 | .85 | .83 | -.02 |
| SPA | SPA1 | .83 | .81 | -.02 |

## Table A5. Comparison of model fit indices between urban and rural samples

| Fit index | Recommended threshold |  | Observed values | |
| --- | --- | --- | --- | --- |
|  |  |  | Urban | Rural |
| **Chi-square (χ²)** | Non-significant preferred |  | 312.5, df = 132, p < .001 | 298.7, df = 132, p < .001 |
| **CFI (comparative fit index)** | ≥ .90 (good), ≥ .95 (excellent) |  | .952 | .948 |
| **TLI (Tucker-Lewis index)** | ≥ .90 (good), ≥ .95 (excellent) |  | .945 | .941 |
| **RMSEA (root mean square error of approx.)** | ≤ .08 (acceptable), ≤ .05 (good) |  | .041 | .043 |
| **SRMR (standardized root mean square residual)** | ≤ .08 |  | .038 | .041 |

**Notes:** χ² is sensitive to large sample sizes (N = 706 urban, N = 498 rural); therefore, **incremental and absolute fit indices (CFI, TLI, RMSEA, SRMR)** were prioritized in evaluating model adequacy.

## Table A6. Cross-loadings of measurement items by urban and rural samples

| Item code | Measurement item/indicator | Rural | | | Urban | | |
| --- | --- | --- | --- | --- | --- | --- | --- |
|  |  | SES | SPA | MHU | SES | SPA | MHU |
| MHU1 | Using mHealth applications motivates me to maintain regular physical activity. | .37 | .46 | **.85** | .39 | .48 | **.87** |
| MHU2 | Using mHealth applications for managing my body weight and physical activity is a pleasant experience. | .35 | .44 | **.87** | .37 | .46 | **.89** |
| MHU3 | Using mHealth applications helps me monitor and manage my physical activity more effectively. | .33 | .42 | **.86** | .35 | .44 | **.88** |
| MHU4 | I regularly spend time using mHealth applications to track my physical activity or body weight. | .31 | .40 | **.84** | .33 | .42 | **.86** |
| MHU5 | Using mHealth applications allows me to access my health and physical activity information quickly. | .34 | .43 | **.88** | .36 | .45 | **.90** |
| MHU6 | I frequently use mHealth applications to support my physical activity or weight control. | .32 | .41 | **.85** | .34 | .43 | **.87** |
| SPA1 | I engage in physical activity regularly every week. | .39 | **.86** | .45 | .41 | **.88** | .47 |
| SPA2 | I maintain a consistent routine of physical activity. | .37 | **.88** | .44 | .39 | **.90** | .46 |
| SPA3 | I allocate sufficient time for physical activity in my daily or weekly schedule. | .35 | **.85** | .42 | .37 | **.87** | .44 |
| SPA4 | I continue to perform physical activities even when I am busy. | .36 | **.84** | .41 | .38 | **.86** | .43 |
| SPA5 | I try to maintain physical activity as part of my lifestyle. | .38 | **.87** | .43 | .40 | **.89** | .45 |
| SPA6 | Degree of physical activity during leisure and transport over the past year. | .34 | **.82** | .39 | .36 | **.84** | .41 |
| SES1 | Education attainment | **.83** | .37 | .34 | **.85** | .39 | .36 |
| SES2 | Household income | **.86** | .39 | .35 | **.88** | .41 | .37 |
| SES3 | Employment stability | **.84** | .36 | .33 | **.86** | .38 | .35 |

Note: MHU = mHealth utilization (6 items: MHU1-MHU6), SPA=sustained physical activities (6 items: SPA1-SPA6), SES – socio-economic status (3 items: SES1-SES3).
